# A Genetic Risk Score for Recurrent Miscarriages Based on Polymorphisms in Platelet Glycoproteins and Adhesion Molecules Genes

**DOI:** 10.3390/jcm14072355

**Published:** 2025-03-29

**Authors:** Nikolaos Vlachadis, Chryssi Christodoulaki, Vassilios Tsamadias, Panagiotis Peitsidis, Nikolaos Machairiotis, Dimos Sioutis, Nikolaos F. Vlahos, Emmanuel Economou, Periklis Panagopoulos

**Affiliations:** 1 Department of Obstetrics and Gynecology, General Hospital of Messinia, 24100 Kalamata, Greece; vlaxadis@gmail.com; 2 Clinical Laboratory of Genetic-Therapeutic Individualization, Second Department of Obstetrics and Gynecology, Medical School, Aretaieio Hospital, National and Kapodistrian University of Athens, 11528 Athens, Greece; vtsam5735@gmail.com (V.T.); eveconom@pharmagenetix.net (E.E.); 3 Third Department of Obstetrics and Gynecology, Medical School, Attiko Hospital, National and Kapodistrian University of Athens, 12462 Athens, Greece; christodoulakichr@hotmail.com (C.C.); nikolaosmachairiotis@gmail.com (N.M.); dsioutis@gmail.com (D.S.); 4 Fifth Department of Obstetrics and Gynecology, Elena Venizelou Maternity Hospital, 11521 Athens, Greece; 5 Second Department of Obstetrics and Gynecology, Medical School, Aretaieio Hospital, National and Kapodistrian University of Athens, 11528 Athens, Greece; nfvlahos@gmail.com; 6 Family Planning Unit, Third Department of Obstetrics and Gynecology, Medical School, Attiko Hospital, National and Kapodistrian University of Athens, 12462 Athens, Greece; paninosrafaela@yahoo.gr

**Keywords:** miscarriage, recurrent miscarriage, recurrent pregnancy loss, polymorphism, single nucleotide polymorphism (SNP), platelets, thrombophilia, platelet glycoproteins, genetic risk score

## Abstract

**Background/Objectives:** The objective of the study was to explore the combined effect of polymorphisms in the platelet glycoproteins Ia (GpIa) and IIIa (GpIIIa), along with the platelet-endothelial cell adhesion molecule-1 (PECAM-1) and P-Selectin genes, on the risk of recurrent pregnancy loss. **Methods:** This study involved 162 women with primary unexplained recurrent miscarriages and 60 fertile controls who had at least one uncomplicated full-term pregnancy without experiencing fetal loss. All participants were of Greek origin and were genotyped for four single nucleotide polymorphisms (SNPs), GpIa-C807T, GpIIIa-PlA1/PlA2, PECAM-1-C373G, and P-Selectin-A37674C, using pyrosequencing. A genetic risk score (GRS) was calculated in two forms: one based on the number of SNPs (dominant model) and the other based on the number of polymorphic alleles (additive model), utilizing logistic regression and receiver operator characteristic (ROC) analyses. **Results:** A statistically significant increase in the risk of miscarriage was observed with the number of polymorphic genes, with an odds ratio (OR) of 2.2 (95% confidence interval [CI]: 1.5 to 3.2, *p* < 0.001) for each additional SNP. The ROC analysis revealed an area under the curve (AUC) of 0.689 (95% CI: 0.614 to 0.763, *p* < 0.001). The presence of two or more polymorphic genes demonstrated a sensitivity of 69.8% and specificity of 65%, with an OR = 4.3 (95% CI: 2.3 to 8.0, *p* < 0.001). The performance of the GRS improved in younger patients and those experiencing late miscarriages. An AUC = 0.839 (95% CI: 0.749 to 0.930, *p* < 0.001) and an OR = 7.0 (95% CI: 2.8 to 17.8, *p* < 0.001) per SNP were achieved for the age group < 30 years. For subjects with second trimester fetal loss, the GRS yielded an AUC = 0.742 (95% CI: 0.610 to 0.874, *p* = 0.002) and an OR = 3.6 (95%OR = 7.0, 95% CI: 2.8 to 17.8) per SNP. The allelic GRS produced similar or slightly diminished results. **Conclusions:** This study highlights the promising potential of a genetic risk score based on four SNPs in predicting unexplained recurrent miscarriages, particularly in younger individuals and in cases of late miscarriage. These findings contribute to a deeper understanding of the epidemiology of unexplained recurrent miscarriage, emphasizing the role of platelet thrombophilia.

## 1. Introduction

While sporadic miscarriages are a common complication of pregnancy, recurrent miscarriages are classified as a distinct condition characterized by the occurrence of two or more clinical pregnancy losses before 20 weeks’ gestation, affecting approximately 2% of women [1,2]. Recurrent pregnancy loss can be categorized as primary when there are multiple miscarriages without a live birth, or secondary when the woman has experienced a successful pregnancy in addition to recurrent miscarriages. Recurrent fetal loss is a profoundly distressing medical condition that can have serious emotional consequences for couples and significantly impact their fertility [2,3]. Despite comprehensive investigations and the identification of various potential causative factors, the underlying etiology of this syndrome remains unclear in at least 50% of cases [1,2,3].

A plethora of studies have reported that thrombophilic gene variants, which are associated with an increased risk of developing abnormal blood clots, may also be linked to an increased incidence of miscarriage by interfering with normal blood flow to the placenta and developing fetus [4,5,6]. The thrombophilic gene polymorphisms most extensively studied in relation to miscarriage risk include genetic variations in specific factors implicated in blood coagulation and fibrinolysis, such as factor V Leiden, prothrombin (factor II), and plasminogen activator inhibitor-1 (PAI-1), as well as mutations that result in alterations to proteins C, S, and homocysteine metabolism [4,5,6].

Glycoproteins Ia (GpIa) and IIIa (GpIIIa), and adhesion molecules P-selectin, and PECAM-1 (platelet endothelial cell adhesion molecule) are membrane-bound molecules on the surface of platelets, with critical roles in platelet adhesion, aggregation, and thrombosis [7,8]. GpIa is a component of the platelet receptor complex GpIa/IIa, necessary for platelet adhesion to subendothelial collagen and activation. GpIIIa is involved in the formation of receptor complexes GpIIb/IIIa and GpV/IIIa and plays a crucial role in platelet aggregation and thrombus stabilization through binding to fibrinogen or von Willebrand factor. The GpIa-C807T (rs1126643) single nucleotide polymorphism (SNP) is linked to changes in the density of GpIa/IIa receptors, leading to increased collagen binding. Similarly, the GpIIIa-T1565C-PlA1/PlA2 (rs5918) genetic polymorphism causes structural changes in the receptor, resulting in heightened adhesive activity. Both SNPs have been reported to increase the risk of arterial thrombosis [9,10].

P-selectin and PECAM-1 are involved in the regulation of platelet aggregation and the complex interactions between activated platelets, leukocytes, and the endothelium. Together, these molecules are particularly crucial in the development of thromboinflammatory responses and contribute to the progression of pathological thrombosis [7]. PECAM-1-C373G (Leu125Val) (rs668) and P-Selectin-A37674C (Thr715Pro) (rs6136) genetic polymorphisms have been epidemiologically linked to the risk of cardiovascular thrombotic events [11,12].

Recently, we explored the genetic associations of these SNPs with the risk of recurrent unexplained miscarriages. Our findings revealed that these associations were particularly significant for individuals carrying combinations of the identified SNPs [12,13]. Building on these findings, this study aimed to further investigate the collective impact of these four SNPs on the risk of recurrent pregnancy loss. By establishing a genetic risk score (GRS), we sought to provide a predictive tool for assessing the likelihood of recurrent pregnancy loss based on an individual’s genetic profile.

## 2. Materials and Methods

This is a secondary analysis of the results from a case-control study conducted at Aretaieio University Hospital, Athens, Greece. The characteristics of the participants in both the study and control groups, as well as the DNA genotyping procedures, have been thoroughly detailed in previous reports [12,13]. In summary, this study included 162 nulligravida women aged 24 to 40 years who had experienced at least two miscarriages, along with 60 fertile controls. All participants were genotyped for four SNPs: GpIa-C807T, GpIIIa-PlA1/PlA2, PECAM-1-C373G, and P-Selectin-A37674C, using pyrosequencing. Women in the study group were selected after excluding all known risk factors for recurrent miscarriages, including anatomic, chromosomal, hormonal, immunologic, or infectious causes. Sixty healthy women over the age of 40, who have had least one uncomplicated full-term pregnancy and who have not experienced fetal loss, served as controls. All participants were of Greek descent.

The study project was conducted in three stages. Firstly, genetic polymorphisms GpIa-C807T and GpIIIa-PlA1/PlA2 were examined individually and in combination to determine their impact on the risk of recurrent miscarriage [13]. Secondly, the associations between PECAM-1-C373G (Leu125Val) and P-Selectin-A37674C (Thr715Pro) SNPs and the risk of recurrent miscarriages were investigated [12]. This study involves a secondary analysis of all available data to study the cumulative effect of these four SNPs through a GRS on the risk of recurrent miscarriages.

Quantitative variables were presented as mean ± standard deviation (SD) after confirming normal distribution using D’Agostino–Pearson’s test, and means were compared using a two-sample Student’s *t*-test. Frequencies were compared using Pearson’s chi-square test and Fisher’s exact test when appropriate. The strength of each association was reported as an odds ratio (OR) with 95% confidence intervals (95% CI). The Haldane–Anscombe correction was applied to calculate the OR when any cell in the contingency table contained a zero.

A GRS was calculated in two forms. First, it was based on the number of SNPs, i.e., the number of genes carrying at least one polymorphic allele, representing a dominant model. This ranged from 0 (if all gene loci are homozygous for the low-risk allele) to 4 (if all gene loci have at least one polymorphic allele). In the second form, the GRS was calculated based on the total number of polymorphic alleles (GpIa-807T, GpIIIa-PlA2, PECAM-1-373G, and P-Selectin-37674C) carried by each participant. This was an additive model, meaning that the more polymorphic alleles a person has across all the genes, the higher the risk of miscarriages, reflecting the cumulative effect of these alleles. In this model, the GRS ranged from 0 (if all alleles are wild type) to 8 (if all gene loci are homozygous for the polymorphic allele). The GRS was initially used to analyze the entire case population and was then further investigated with respect to maternal age, focusing on those aged < 35 years (median) (n = 84) and the youngest age group (<30 years) (n = 21). Additionally, the GRS was assessed based on the gestational age at which fetal loss occurred. Specifically, 58 women who experienced at least one miscarriage at ≥10 weeks of gestation (fetal period) were classified as having “late miscarriage”, while 19 participants who had at least one pregnancy loss after 12 weeks were categorized as having “second trimester miscarriage”.

Logistic regression models were applied, and the OR for miscarriages per SNP (dominant model) and per polymorphic allele (additive model) were calculated as the exponentiation of the coefficient beta. The performance of the GRS in identifying women with recurrent miscarriage was evaluated using receiver-operating characteristic (ROC) analysis, where the area under the curve (AUC) was calculated, along with sensitivity and specificity at the optimal cut-off point. Statistical significance was defined as a *p*-value < 0.05 (two-sided). The analysis was conducted using MedCalc statistical software, version 12.7.

## 3. Results

**Figure 1:**
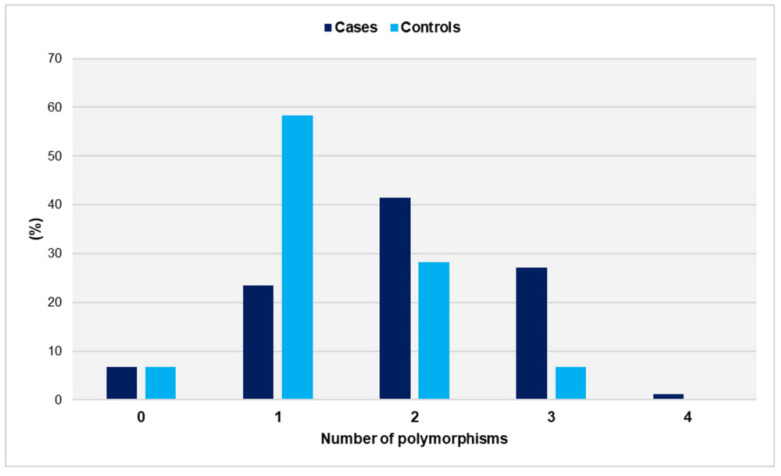
Distribution of genetic polymorphisms GpIa-C807T, GpIIIa-PlA1/PlA2, PECAM-1-C373G, and P-Selectin-A37674C in women with miscarriages (cases) and fertile women (controls).

The distribution of genetic polymorphisms in women with miscarriages and fertile women is shown in Figure 1.

The number of polymorphisms ranged from zero to four in the study group and from zero to three in the controls. Notably, no control carried all four SNPs. The distributions did not differ significantly from the normal distribution (*p* = 0.097 for cases and *p* = 0.158 for controls). Women with a history of unexplained spontaneous abortions had a significantly higher number of SNPs compared with the control group (mean ± SD: 1.9 ± 0.9 vs. 1.3 ± 0.7, *p* < 0.001). Compared with women carrying ≤ 1 SNP, carriers of two SNPs had a significantly increased relative risk of miscarriage (OR = 3.1, 95% CI: 1.6 to 6.2, *p* < 0.001). The risk was further increased for carriers of polymorphic alleles in at least three gene loci (OR = 9.2, 95% CI: 3.0 to 27.6, *p* < 0.001) (Table 1).

**Figure 2:**
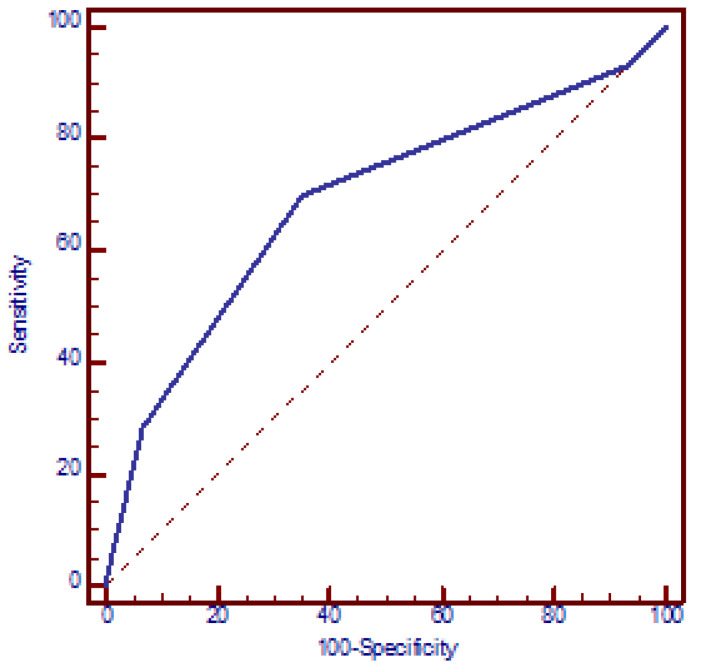
Receiver operating characteristic (ROC) curve analysis assessing the discriminatory power of the number of genetic polymorphisms in relation to recurrent miscarriages. AUC = 0.689 (95% CI: 0.614 to 0.763, *p* < 0.001). At a cut-off value of two polymorphic genes, the model yielded a sensitivity of 69.8% (95% CI: 65.5% to 73.5%) and a specificity of 65.0% (95% CI: 53.5% to 75.2%).

The increased risk of miscarriages with the number of SNPs was confirmed using a logistic regression model. According to this model, the presence of each additional SNP resulted in a mean 2.2-fold increase in the OR for miscarriages (95% CI: 1.5 to 3.2, *p* < 0.001) (beta = 0.78). The discriminatory power of the four polymorphic genes in identifying women with miscarriages was assessed using an ROC curve, which yielded an AUC of 0.689 (95% CI: 0.614 to 0.763, *p* < 0.001). The presence of ≥2 polymorphic genes exhibited the best functional characteristics, with a sensitivity of 69.8% (95% CI: 65.5% to 73.5%) and specificity of 65.0% (95% CI: 53.5% to 75.2%) and OR = 4.3 (95% CI: 2.3 to 8.0, *p* < 0.001) (Figure 2).

**Figure 3:**
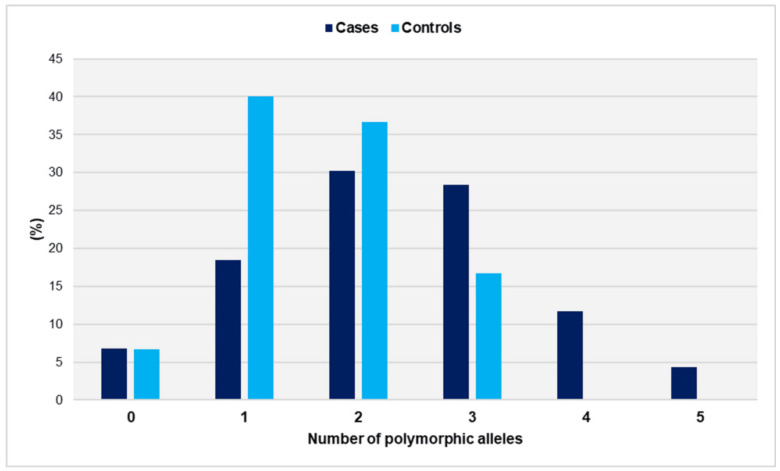
Distribution of polymorphic alleles GpIa-807T, GpIIIa-PlA2, PECAM-1-373G, and P-Selectin-37674C in women with miscarriages (cases) and fertile women (controls).

The distribution of polymorphic alleles in cases and controls is depicted in Figure 3.

The range of polymorphic alleles varied from zero to five in the cases and from zero to three in the controls. It is worth noting that none of the controls carried more than three risk alleles. The distributions did not show a significant deviation from the normal distribution (*p* = 0.466 for cases and *p* = 0.407 for controls). The number of polymorphic alleles was statistically significantly higher in women who had experienced unexplained spontaneous miscarriages compared with controls (mean ± SD: 2.3 ± 1.2 vs. 1.6 ± 0.8, *p* < 0.001). In comparison with women with ≤1 polymorphic allele, those carrying three polymorphic alleles had an OR of 3.1 (95% CI: 1.4 to 7.2, *p* = 0.006). The risk of recurrent miscarriage was significantly elevated for women carrying ≥ 4 polymorphic alleles (OR = 36.4, 95% CI: 2.1 to 621.9, *p* = 0.013). The presence of two polymorphic alleles was associated with a non-significantly increased relative risk (OR = 1.5, 95% CI: 0.8 to 3.0, *p* = 0.237) (Table 2).

**Figure 4:**
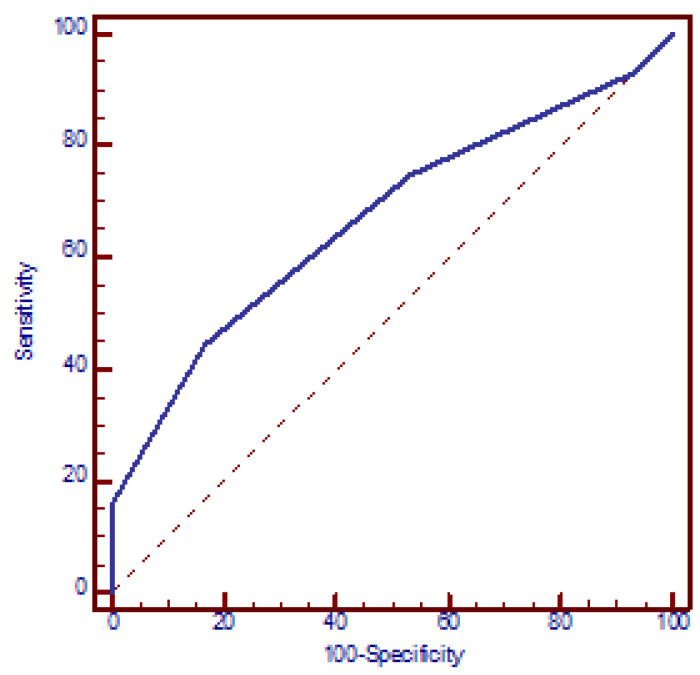
Receiver operating characteristic (ROC) curve analysis assessing the discriminatory power of the number of polymorphic alleles in relation to recurrent miscarriages. AUC = 0.669 (95% CI: 0.603 to 0.731, *p* < 0.001). At a cut-off value of three polymorphic alleles, the model yielded a sensitivity of 44.4% (95% CI: 36.6% to 52.4%) and a specificity of 83.3% (95% CI: 71.5% to 91.7%).

The logistic regression model demonstrated a significant correlation between the risk of miscarriages and the number of polymorphic alleles, with each additional allele increasing the OR by a factor of 1.8 (95% CI: 1.3 to 2.3, *p* < 0.001) (beta = 0.56). The ROC analysis yielded an AUC of 0.669 (95% CI: 0.603 to 0.731, *p* < 0.001). The optimal discriminative ability was observed at a cut-off of three polymorphic alleles, with a sensitivity of 44.4% (95% CI: 36.6% to 52.4%) and a specificity of 83.3% (95% CI: 71.5% to 91.7%) and OR = 4 (95% CI: 1.9 to 8.4, *p* < 0.001) (Figure 4).

The GRS was then applied by maternal age. For women aged <35 years, carriers of ≥3 versus ≤1 polymorphic gene had an OR = 13.3 (95% CI: 4.1 to 43.72, *p* < 0.001), and carriers of two SNPs had an OR = 4.7 (95% CI: 2.1 to 10.4, *p* < 0.001). These associations were strongest among women aged <30 years, with OR = 27.5 (95% CI: 3.3 to 228.9, *p* < 0.001) for carriers of one combination of SNPs, and OR = 78 (95% CI: 7.7 to 793.4, *p* < 0.001) for those with ≥3 SNPs (Table 3 and Table 4).

Furthermore, in the <35 age group, compared with individuals carrying ≤1 risk allele, those carrying three and ≥4 polymorphic alleles displayed ORs of 4.3 (95% CI: 1.7 to 11.0, *p* < 0.001) and 47.2 (95% CI: 2.7 to 842.5, *p* < 0.001), respectively. Carriers of two risk alleles showed a non-significant association (OR = 2.0, 95% CI: 0.9 to 4.6, *p* = 0.103). Notably, the elevated relative risks observed were more pronounced among women under the age of 30, with ORs of 22.4 (95% CI: 2.5 to 202.3, *p* < 0.001) and 247 (95% CI: 9.0 to 6778.4, *p* < 0.001) for carriers of three and ≥4 polymorphic alleles, respectively. A nearby correlation to statistical significance was also found for carriers of two alleles (OR = 7.6, 95% CI: 1.0 to 68.2, *p* = 0.052) (Table 5 and Table 6).

The GRS was subsequently evaluated in relation to the gestational age of miscarriage. For miscarriage at ≥10 weeks’ gestation, the OR was 2.7 (95% CI: 1.2 to 6.3, *p* = 0.021) and 10.3 (95% CI: 3.1 to 34.7, *p* < 0.001) for carriers of two SNPs and carriers of ≥3 SNPs compared with those with ≤1 SNP, respectively. For fetal loss after 12 weeks, the OR for carriers of two polymorphic genes was marginally statistically non-significant at 3.2 (95% CI: 0.9 to 11.6, *p* = 0.096), but it reached 13.7 (95% CI: 2.9 to 63.8, *p* < 0.001) for carriers of ≥3 genetic polymorphisms (Table 7 and Table 8).

Further, compared with women with ≤1 risk allele, the risk of miscarriage at >10 weeks was statistically non-significantly increased for carriers of two risk alleles (OR = 1.5, 95% CI: 0.6 to 3.8, *p* = 0.342), and rose further for carriers of three and ≥4 risk alleles, with an OR = 3 (95% CI: 1.1 to 8.4, *p* = 0.033) and OR = 49.1 (95% CI: 2.7 to 890.0, *p* = 0.008), respectively. For second-trimester miscarriage, the relative risk was statistically non-significantly elevated for carriers of two and three risk alleles (OR = 1.6, 95% CI: 0.4 to 6.6, *p* = 0.719, and OR = 2.8, 95% CI: 0.6 to 13.4, *p* = 0.222, respectively), rising to an OR of 82.3 (95% CI: 3.9 to 1727.2, *p* = 0.004) for those carrying ≥4 polymorphic alleles (Table 9 and Table 10).

Logistic regression indicated a significant rise in the relative risk of miscarriages as the number of polymorphic genes and alleles increased. The OR for miscarriages was 2.8 per SNP for individuals < 35 years (95% CI: 1.8 to 4.5, *p* < 0.001) and higher for those <30 years (OR = 7.0, 95% CI: 2.8 to 17.8, *p* < 0.001), as well as for late miscarriages (≥10 weeks: OR = 2.5, 95% CI: 1.6 to 4.1, *p* < 0.001; > 12 weeks: OR = 3.6, 95% CI: 1.7 to 7.5, *p* < 0.001). Additionally, the risk of spontaneous abortions (per allele) was more prominent in younger patients (<35 years: OR = 2.1, 95% CI: 1.4 to 3.0, *p* < 0.001; <30 years: OR = 5.3, 95% CI: 2.4 to 11.7, *p* < 0.001) and at more advanced gestational age (≥10 weeks: OR = 2.0, 95% CI: 1.4 to 3.0, *p* < 0.001; second trimester: OR = 2.8, 95% CI: 1.6 to 5.1, *p* < 0.001) (Table 11 and Table 12).

The ROC analysis of the GRS based on the number of SNPs resulted in an AUC of 0.729 (95% CI: 0.645 to 0.812, *p* < 0.001) for women < 35 years, with excellent performance observed in the youngest age group (< 30 years) (AUC = 0.839, 95% CI: 0.749 to 0.930, *p* < 0.001). Sensitivity increased to 77.4% (95% CI: 70.2% to 83.6%) at ages < 35 years and further to 95.2% (95% CI: 76.7% to 99.8%) in women aged < 30 years when using a cut-off of two SNPs, and the OR was 6.4 (95% CI: 3.0 to 13.3, *p* < 0.001) and 37.1 (95% CI: 4.7 to 296.5, *p* < 0.001), respectively. The AUC was 0.698 for miscarriage ≥ 10 gestational weeks (95% CI: 0.603 to 0.794, *p* < 0.001) and 0.742 (95% CI: 0.610 to 0.874, *p* = 0.002) for second trimester fetal loss. Carrying ≥2 SNPs had a sensitivity of 69.0% (95% CI: 59.0% to 77.7%) with an OR = 4.1 (95% CI: 1.9 to 8.9, *p* < 0.001), and 73.7% (95% CI: 51.5% to 89.3%) with an OR = 5.2 (95% CI: 1.7 to 16.4, *p* = 0.003) for miscarriage at ≥10 weeks’ and >12 weeks’ gestation, respectively (Table 13).

In a similar vein, the ROC analysis of the GRS based on the number of risk alleles resulted in an AUC of 0.699 (95% CI: 0.615 to 0.784, *p* < 0.001) for subjects aged < 35 years and 0.833 (95% CI: 0.734 to 0.933, *p* < 0.001) for those < 30 years. Using a cut-off of three polymorphic alleles, the sensitivity increased from 47.6% (95% CI: 40.7% to 52.9%) to 66.7% (95% CI: 46.8% to 82.3%) for these age groups, with an OR = 4.5 (95% CI: 2.0 to 10.1, *p* < 0.001) and OR = 10 (95% CI: 3.2 to 31.0, *p* < 0.001), respectively. For miscarriages at ≥10 weeks and >12 weeks, the AUC was 0.687 (95% CI: 0.590 to 0.783, *p* < 0.001) and 0.736 (95% CI: 0.596 to 0.876, *p* = 0.002), respectively. The presence of ≥3 polymorphic alleles had a sensitivity of 68.7% (95% CI: 59.0% to 78.3%) for fetal loss at ≥10 weeks of gestation and 73.6% (95% CI: 51.5% to 89.3%) for second trimester pregnancy loss, with an OR = 4.4 (95% CI: 1.9 to 10.2, *p* < 0.001) and OR = 5.6 (95% CI: 1.8 to 17.2, *p* = 0.003), respectively (Table 14).

## 4. Discussion

In the present study, the combined effect of four genetic polymorphisms associated with platelet-related thrombophilia on the risk of recurrent pregnancy loss was investigated. Two of these SNPs are linked to platelet glycoprotein genes, while the other two are associated with adhesion molecule genes. Using a case-control design, we successfully derived a GRS that demonstrated a remarkable ability to predict recurrent fetal loss.

We previously reported statistically significant associations between miscarriages and the presence of the GpIa-807T and GpIIIa-PlA2 alleles, with an especially heightened risk for combined carriers [13]. In a subsequent publication, a significantly increased risk of pregnancy loss was observed for the combination of the PECAM-1 (Leu125Val) and P-Selectin (Thr715Pro) SNPs [12]. This was a secondary analysis examining the simultaneous effects of the four SNPs, which led to strong associations. Compared with women carrying ≤ 1 SNP, the relative risk of miscarriage was increased for carriers of two SNPs (OR = 3.1) and even higher for carriers of ≥3 SNPs (OR = 9.2). Additionally, compared with subjects carrying ≤ 1 polymorphic allele, the risk appeared to increase with the number of polymorphic alleles, reaching an OR = 36.4 for carriers of ≥4 risk alleles.

The logistic regression analysis revealed a statistically significant multiplicative increase in the odds of miscarriage for each additional SNP (OR = 2.2) and for each polymorphic allele (OR = 1.8). Furthermore, the ROC analysis demonstrated an AUC of 0.689, indicating a good ability to accurately classify women with recurrent fetal loss. A cut-off of two SNPs resulted in a sensitivity of 69.8% with an OR of 4.3. The discriminatory ability was slightly diminished for the allelic form of the GRS, with an AUC of 0.669.

The GRS was stratified by maternal age, revealing stronger associations and increased relative risks per SNP for the younger half of the age distribution of cases (those < 35 years). The relative risks were even higher for the subgroup aged <30 years. The area under the curve (AUC) improved to 0.729 for women < 35 years and reached an excellent 0.839 for those < 30 years (0.833 for the allelic GRS). Similarly, in the analysis based on gestational age at miscarriage, the ORs were elevated for the subgroup with losses at ≥10 weeks, and even higher for those occurring in the second trimester.

In this study, an evaluation was conducted on a GRS focusing on four platelet thrombophilic genes. The results indicated a significant increase in the risk of miscarriage as the number of polymorphic genes and alleles increased, both in a dominant and additive perspective. These findings suggest a synergistic effect of these genes on the risk of pregnancy loss. The underlying biological mechanisms are likely related to the sequential interaction of the four receptors encoded by these genes in the formation of blood clots. Specifically, GpIa is involved in the initial activation of platelets and their binding to collagen, GpIIIa plays a crucial role in platelet aggregation, while PECAM-1 and P-Selectin contribute to platelet adhesion and activation, facilitating the interaction of activated platelets with leukocytes and the endothelium [7,8,14]. The significance of the genetic polymorphisms GpIa-C807T and GpIIIa-PlA1/PlA2 has been demonstrated phenotypically in a study evaluating platelet aggregation through the measurement of collagen/epinephrine closure time (COL/EPI CT). The study found that carriers of these SNPs, particularly those who carried both risk variants, exhibited enhanced platelet aggregation, which could play a critical role in impaired perfusion during the early stages of gestational development [15]. It is also noteworthy that glycoproteins Ia (integrin alpha 2) and IIIa (integrin beta 3), along with the adhesion molecules PECAM-1 and P-Selectin, are present in the receptive endometrium and human trophoblast and actively involved in the processes of embryo implantation as well as the early stages of successful pregnancy progression, including placentation and angiogenesis [16,17,18].

Furthermore, our analysis by maternal age revealed that younger women, particularly those under the age of 30, had a higher relative risk of miscarriages associated with the presence of multiple genetic polymorphisms. This finding adds further biological validity to the association of these polymorphisms with pregnancy loss. Since it is known that the main causative factor of miscarriages is chromosomal defects that increase with maternal age, other risk factors are expected to emerge stronger at younger ages where the incidence of miscarriages due to chromosomal abnormalities is expected to be lower [1,3,19].

Additionally, our study found that the risk of miscarriage was more pronounced at later gestational ages, with carriers of multiple genetic polymorphisms showing a significantly higher relative risk of miscarriage at ≥ 10 weeks and after 12 weeks of gestation. This finding may be interpreted considering the higher incidence of chromosomal defects in early miscarriages and the enhanced role of thrombophilia in the pathogenesis of late pregnancy losses, particularly after placental formation is complete (fetal period). In this regard, Ivanov et al. reported statistically significant associations between recurrent miscarriage and GpIIIa-PlA2 allele carriage, with the relative risk being higher between 10 and 20 weeks than before 10 weeks of gestation [20]. Furthermore, a series of studies have shown that Factor V Leiden presents a higher relative risk for late fetal loss [21]. In the present study, pregnancies lost in the second trimester were classified as low risk for chromosomal abnormalities after standard first-trimester screening. Moreover, previous reports indicate that the associations of individuals carrying both the GpIa-T and GpIIIa-PlA2 alleles, as well as those with the PECAM-1-373G and P-Selectin-37674C alleles, are stronger in cases of late fetal loss [12,21].

We obtained an AUC of 0.689 for the overall cases population using only four SNPs. Notably, our GRS performed significantly better in younger patients, with a value of 0.839 for ages < 30 years. Additionally, the GRS showed improved discriminative ability for late miscarriages. These results were consistent when applying the allelic form of our GRS. A previous study tested a GRS consisting of 12 SNPs from various coagulation, hormonal, endometrial, and inflammatory genes in a Ukrainian population sample, yielding an AUC of 0.64 for predicting the risk of recurrent pregnancy loss [22]. In a separate study in Spain, a thrombophilic GRS was created using 12 SNPs along with maternal age, resulting in an AUC of 0.763 [23]. However, in this study, a risk score that was purely genetic and did not include maternal age was utilized to avoid scenarios in which a patient with only a limited number of thrombophilic variants could be erroneously categorized as high risk simply due to her older age. Instead, we applied the GRS separately in different age groups, revealing the biologically plausible improved performance of the GRS in younger women.

These four SNPs have also been investigated for their role in recurrent implantation failure (RIF) following in vitro fertilization (IVF). It was observed that the presence of the GpIa-807T and GpIIIa-PlA2 alleles, as well as the simultaneous carriage of the PECAM-1-373G and P-Selectin-37674C alleles, are associated with an increased risk of RIF, particularly in younger patients. Eventually, the combined analysis of these genetic variations showed a significant association with the risk of RIF, especially in younger women, and produced a genetic risk score that demonstrated good diagnostic accuracy in predicting RIF (AUC = 0.754 for the SNP GRS and AUC = 0.725 for the allelic GRS) [24]. This likely indicates that shared disease-causing mechanisms related to the four polymorphic alleles are involved in both RIF and recurrent pregnancy loss.

Recurrent miscarriage is a complex disorder influenced by multiple etiological factors. Inherited thrombophilia has been associated with fetal loss, and evidence suggests that a combination of variants serves as a more effective diagnostic tool since individual genetic polymorphisms typically contribute only modestly to the overall risk within the population [25,26]. We developed a GRS that showed strong predictive performance, particularly in younger individuals and in cases of late fetal loss. The success of this GRS was largely due to the careful selection of candidate genetic variants that had been evaluated in earlier stages of our research, as well as the stringent criteria used to select patients with primary recurrent miscarriages and the corresponding controls. A key limitation of our study is the relatively small sample size. Overall, our findings suggest that genetic polymorphisms in platelet glycoproteins and cell adhesion molecule genes may serve as valuable tools for assessing the risk of recurrent miscarriages.

## 5. Conclusions

We present a GRS based on four SNPs, which demonstrated good discriminatory ability in predicting unexplained recurrent miscarriage. The results showed that the risk of fetal loss increased with the number of polymorphic genes or alleles. The GRS exhibited enhanced performance in younger individuals and in cases of late miscarriage. These findings offer new insights into the etiology of unexplained recurrent miscarriage, highlighting the role of platelet thrombophilia in the development of the disorder. They also likely identify a group of women who may benefit significantly from prophylactic antiplatelet therapy. Further research is needed to confirm the GRS’s performance in larger sample sizes and to explore its potential enhancement with additional genetic variants, ultimately aiming to develop personalized approaches for the prevention and anticoagulation management of unexplained recurrent pregnancy loss.

## Figures and Tables

**Table 1:** Associations between miscarriages and genetic risk score based on the number of genetic polymorphisms.

Number of Genetic Polymorphisms	Cases		Controls		Odds Ratio	95% Confidence Interval	*p*-Value
	n	(%)	n	(%)			
≥3	46	28.4	4	6.7	9.2	3.0 to 27.6	<0.001
2	67	41.4	17	28.3	3.1	1.6 to 6.2	<0.001
≤1	49	30.2	39	65	Reference		

**Table 2:** Associations between miscarriages and genetic risk score based on the number of polymorphic alleles.

Number of Polymorphic Alleles	Cases		Controls		Odds Ratio	95% Confidence Interval	*p*-Value
	n	(%)	n	(%)			
≥4	26	16.0	0	0	36.4	2.1 to 621.9	0.013
3	46	28.4	10	16.7	3.1	1.4 to 7.2	0.006
2	49	30.2	22	36.7	1.5	0.8 to 3.0	0.237
≤1	41	25.3	28	46.7	Reference		

**Table 3:** Associations between miscarriages and genetic risk score based on the number of genetic polymorphisms (<35 years age group).

Number of Genetic Polymorphisms	Cases		Controls		Odds Ratio	95% Confidence Interval	*p*-Value
	n	(%)	n	(%)			
≥3	26	31.0	4	6.7	13.3	4.1 to 43.7	<0.001
2	39	46.4	17	28.3	4.7	2.1 to 10.4	<0.001
≤1	19	22.6	39	65	Reference		

**Table 4:** Associations between miscarriages and genetic risk score based on the number of genetic polymorphisms (<30 years age group).

Number of Genetic Polymorphisms	Cases		Controls		Odds Ratio	95% Confidence Interval	*p*-Value
	n	(%)	n	(%)			
≥ 3	8	38.1	4	6.7	78	7.7 to 793.4	<0.001
2	12	57.1	17	28.3	27.5	3.3 to 228.9	<0.001
≤ 1	1	4.8	39	65	Reference		

**Table 5:** Associations between miscarriages and genetic risk score based on the number of polymorphic alleles (<35 years age group).

Number of Polymorphic Alleles	Cases		Controls		Odds Ratio	95% Confidence Interval	*p*-Value
	n	(%)	n	(%)			
≥4	14	16.7	0	0	47.2	2.7 to 842.5	0.009
3	26	31.0	10	16.7	4.3	1.7 to 11.0	0.003
2	27	32.1	22	36.7	2.0	0.9 to 4.6	0.103
≤1	17	20.2	28	46.7	Reference		

**Table 6:** Associations between miscarriages and genetic risk score based on the number of polymorphic alleles (<30 years age group).

Number of Polymorphic Alleles	Cases		Controls		Odds Ratio	95% Confidence Interval	*p*-Value
	n	(%)	n	(%)			
≥4	6	28.6	0	0	247	9.0 to 6778.4	< 0.001
3	8	38.1	10	16.7	22.4	2.5 to 202.3	< 0.001
2	6	28.6	22	36.7	7.6	1.0 to 68.2	0.052
≤1	1	4.8	28	46.7	Reference		

**Table 7:** Associations between miscarriages and genetic risk score based on the number of genetic polymorphisms (≥10 weeks).

Number of Genetic Polymorphisms	Cases		Controls		Odds Ratio	95% Confidence Interval	*p*-Value
	n	(%)	n	(%)			
≥3	19	32.8	4	6.7	10.3	3.1 to 34.7	<0.001
2	21	36.2	17	28.3	2.7	1.2 to 6.3	0.021
≤1	18	31.0	39	65	Reference		

**Table 8:** Associations between miscarriages and genetic risk score based on the number of genetic polymorphisms (>12 weeks).

Number of Genetic Polymorphisms	Cases		Controls		Odds Ratio	95% Confidence Interval	*p*-Value
	n	(%)	n	(%)			
≥3	7	36.8	4	6.7	13.7	2.9 to 63.8	<0.001
2	7	36.8	17	28.3	3.2	0.9 to 11.6	0.096
≤1	5	26.3	39	65	Reference		

**Table 9:** Associations between miscarriages and genetic risk score based on the number of polymorphic alleles (≥10 weeks).

Number of Polymorphic Alleles	Cases		Controls		Odds Ratio	95% Confidence Interval	*p*-Value
	n	(%)	n	(%)			
≥4	12	20.7	0	0	49.1	2.7 to 890.0	0.008
3	15	25.9	10	16.7	3	1.1 to 8.4	0.033
2	17	29.3	22	36.7	1.5	0.6 to 3.8	0.342
≤1	14	24.1	28	46.7	Reference		

**Table 10:** Associations between miscarriages and genetic risk score based on the number of polymorphic alleles (>12 weeks).

Number of Polymorphic Alleles	Cases		Controls		Odds Ratio	95% Confidence Interval	*p*-Value
	n	(%)	n	(%)			
≥4	6	31.6	0	0	82.3	3.9 to 1727.2	0.004
3	4	21.1	10	16.7	2.8	0.6 to 13.4	0.222
2	5	26.3	22	36.7	1.6	0.4 to 6.6	0.719
≤1	4	21.1	28	46.7	Reference		

**Table 11:** Logistic regression for the association between miscarriages and genetic risk score based on the number of genetic polymorphisms, according to maternal age and gestational age of miscarriage.

	Beta	Odds Ratio	95% Confidence Interval	*p*-Value
Total	0.78	2.2	1.5 to 3.2	<0.001
<35 years	1.04	2.8	1.8 to 4.5	<0.001
<30 years	1.94	7.0	2.8 to 17.8	<0.001
≥10 weeks	0.92	2.5	1.6 to 4.1	<0.001
>12 weeks	1.27	3.6	1.7 to 7.5	<0.001

**Table 12:** Logistic regression for the association between miscarriages and genetic risk score based on the number of polymorphic alleles, according to maternal age and gestational age of miscarriage.

	Beta	Odds Ratio	95% Confidence Interval	*p*-Value
Total	0.56	1.8	1.3 to 2.3	<0.001
<35 years	0.73	2.1	1.4 to 3.0	<0.001
<30 years	1.67	5.3	2.4 to 11.7	<0.001
≥10 weeks	0.70	2.0	1.4 to 3.0	<0.001
>12 weeks	1.04	2.8	1.6 to 5.1	<0.001

**Table 13:** ROC analysis for estimating the discriminatory ability of the number of genetic polymorphisms for the risk of recurrent miscarriages according to maternal age and gestational age of miscarriage. The sensitivity was calculated using a cut-off of two genetic polymorphisms. ROC: Receiver operating characteristic. AUC: Area under the curve.

	AUC	95% Confidence Interval	*p*-Value	Sensitivity (%)	95% Confidence Interval (%)
Total	0.689	0.614 to 0.763	<0.001	69.8	65.5 to 73.5
<35 years	0.729	0.645 to 0.812	<0.001	77.4	70.2 to 83.6
<30 years	0.839	0.749 to 0.930	<0.001	95.2	76.7 to 99.8
>10 weeks	0.698	0.603 to 0.794	<0.001	69.0	59.0 to 77.7
>12 weeks	0.742	0.610 to 0.874	0.002	73.7	51.5 to 89.3

**Table 14:** ROC analysis for estimating the discriminatory ability of the number of polymorphic alleles for the risk of recurrent miscarriages according to maternal age and gestational age of miscarriage. The sensitivity was calculated using a cut-off of three polymorphic alleles. ROC: Receiver operating characteristic. AUC: Area under the curve.

	AUC	95% Confidence Interval	*p*-Value	Sensitivity (%)	95% Confidence Interval (%)
Total	0.669	0.596 to 0.742	<0.001	44.4	40.5 to 47.3
<35 years	0.699	0.615 to 0.784	<0.001	47.6	40.7 to 52.9
<30 years	0.833	0.734 to 0.933	<0.001	66.7	46.8 to 82.3
≥10 weeks	0.687	0.590 to 0.783	<0.001	46.6	37.3 to 54.0
>12 weeks	0.736	0.596 to 0.876	0.002	52.6	32.2 to 71.0

## Data Availability

The raw data supporting the conclusions of this article will be made available by the corresponding author on request.
